# Focal Conic Stacking in Smectic A Liquid Crystals: Smectic Flower and Apollonius Tiling

**DOI:** 10.3390/ma2020499

**Published:** 2009-04-22

**Authors:** Claire Meyer, Loic Le Cunff, Malika Belloul, Guillaume Foyart

**Affiliations:** Laboratoire de Physique des Systèmes Complexes, Université de Picardie Jules Verne, 33 rue Saint-Leu, 80039 Amiens, France; E-Mails: ascorpius@gmail.com (L.C.); malika.belloul@univ-rennes1.fr (M.B.); guillaume.foyart@neuf.fr (G.F.)

**Keywords:** Defects, Focal conic domains, Liquid crystals, Smectic A, Apollonius tiling, Dupin cyclides.

## Abstract

We investigate two different textures of smectic A liquid crystals. These textures are particularly symmetric when they are observed at crossed polars optical microscopy. For both textures, a model has been made in order to examine the link between the defective macroscopic texture and the microscopic disposition of the layers. We present in particular in the case of some hexagonal tiling of circles (similar to the Apollonius tiling) some numeric simulation in order to visualize the smectic layers. We discuss of the nature of the smectic layers, which permit to assure their continuity from one focal conic domain to another adjacent one.

## 1. Introduction

**Figure 1:**
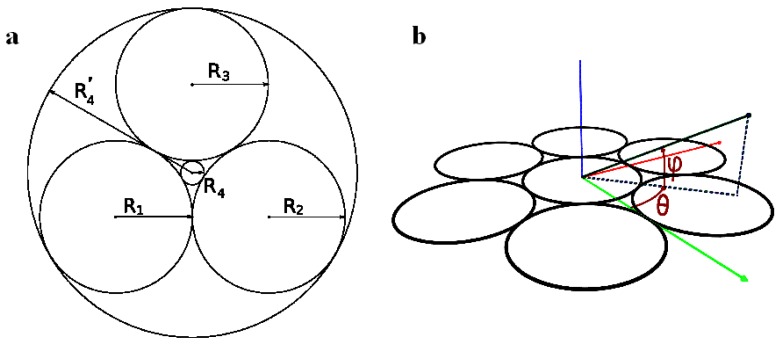
(a): three circles of radii $R_{1}$, $R_{2}$, $R_{3}$ are tangent. The figure has been made in the particular case $R_{1}=R_{2}=R_{3}$. Two circles of Radii $R_{4}$ and $R_{4}^{\prime }$ are tangent to the three initial circles; (b): coordinates system with the two angles, longitude *θ* and latitude *ϕ*.

During many years the problem of the Apollonius tiling has interested a great number of scientists. Many well known mathematicians and physicists have worked on this for several hundred years. These include W. L. Bragg [1], F. Soddy [2], H.S.M. Coxeter [3], R. Descartes [4], P. Beecroft [5], and more recently P. G. De Gennes [6], whose works have a great importance in Soft Matter Physics. René Descartes, in a letter of November 1643 to Princess Elisabeth of Bohemia, developed a formula relating the radii of four mutually tangent circles (Descartes’s theorem), which is: ddeeff+ddeexx+ddffxx+eeffxx=2deffxx+2deeffx+2deefxx+2ddeffx+2ddefxx+2ddeefx where *d*, *e*, *f* are the radii of the three externally tangent circles and where *x* is the radius of the fourth circle. These notations have been simplified, demonstrated by Coxeter [3] and generalized to the case of internal tangent circles in order to obtain:

(1) $$\frac{1}{R_{4}}=\frac{1}{R_{1}}+\frac{1}{R_{2}}+\frac{1}{R_{3}}\pm 2\sqrt{\frac{1}{R_{1}R_{2}}+\frac{1}{R_{1}R_{3}}+\frac{1}{R_{2}R_{3}}}\;$$

where $R_{1}$, $R_{2}$, $R_{3}$ denote the radii of the three initial circles and $R_{4}$ the radii of the fourth circles (see Figure 1a).

In [6] has been recalled that the radius of the $m^{\prime }$th circle in a subseries of circles is of the form

(2) $$R_{m}≅\frac{L}{{\left(m+m_{0}\right)}^{2}}\;$$

where $m_{0}$ is a numerical constant, depending on the choice of the radius of the first circle and *L* is some macroscopic distance associated with the dimension of the largest circle. The problem of Apollonius has been also examined by Mathematicians like Marcel Berger [7] in the frame of geometrical constructions. Nowadays, some papers continue to examine the high dimensional Apollonius Networks [8].

The aim of this paper is to show the importance in soft matter physics of such well ordered structures. J.-B. Fournier and G. Durand [9] have studied the equilibrium shapes of SmA nucleated inside the isotropic phase of some liquid crystal. A particular part of their paper (part 6) concerns asymptotic equilibrium shapes in the limit of large SmA volumes and they have shown that it must correspond to spheres with a radial Apollonius network of conics. The same Apollonius tiling of FCD has been shown by O. D. Lavrentovich and V. M. Pergamenshchik [10] in SmA films made with octyloxycyanobiphenyl/glycerin (8OCB/glycerin). The temperature is decreased from the nematic phase. In the simplest case of toroidal domains (TFCD), the layers are folded around the circle which bounds the domain base and a straight line passing through the center of the circle. The deformation region is restricted by the cylinder (see figure 10b of reference [10]). A very interesting review of the different organizations of FCDs in smectic liquid crystals has been detailed by Y. Bouligand [11]. The geometric constructions are explained, allowing one to better understand the different rules of FCD’s association (for instance, the law of corresponding cones (l.c.c.) mentioned in the suite of our paper, polygonal textures, smectic layers as Dupin cyclides,...). Recently, C. Blanc and collaborators have published some beautiful observations in lyotropic liquid crystals in the system CPCl/hexanol/brine inside the lamellar phase [12, 13]. The textures in the lamellar phase made by focal conics show different generations of focal conics as a function of the sample thickness. Using capillaries of about 100 microns of thickness, they have obtained evidence for three different generations of focal conics. The first generation is made by focal conics at the apices of some hexagonal tiling. The second generation fill the interspacing between focal conics again by some hexagonal tiling of focal conics of smaller sizes. The third generation is made with the decoration of the previous generations by three focal conics of smaller size. This experimental situation can be compared to the Apollonius tiling with three generations. Note that these FCDs do not completely tile the space with these defects and it is this lack of completeness that has been investigated in [6, 11, 14].

Experiments on TFCDs have been recently carried out in order to explore the internal structure of TFCDs. TFCDs have been confined within a microchannel by Kim and coworkers [15]. They have shown in particular that the formation of TFCDs is very much influenced by the channel depth. Another way to investigate the internal structure of TFCDs consists of the use of AFM study on Smectic A droplets either on coated silicon substrates [16] or on $MoS_{2}$ substrates both with hybrid anchoring conditions (homeotropic anchoring with the air-interface and planar anchoring at the solid substrate) [17, 18]. Optical measurements of the birefringence permit the description of TFCDs in thin films of smectic A deposited on mica in air [19].

In this paper, two different thermotropic liquid crystals have been used and observed in the smectic phases under a polarizing microscope. Mainly two different very regular textures have been obtained: a texture that we decided to call the *flower texture* and another one involving the generation of circles, the *generation texture*. The coordinate system for the generation texture is indicated in Figure 1b.

For both textures, the experimental situations will be described in detail. For the flower texture, some visualization of the geometry of the different ellipses and confocal hyperbolae is presented. For the generation texture, some simulation has been made in order to understand how the layers are inside the sample. The aim is to visualize clearly the organization of the smectic layers which take the form of Dupin cyclides. A precise examination of the association of focal conic domains is needed. Note that these Dupin cyclides are useful in a great number of physical situations *i.e.* not only in the physics of liquid crystals, for more information, see [20].

## 2. Materials and Experimental Setup

Two smectic A phases from two different materials have been used. One of them belongs to the cyano-biphenyl series: 8CB (4-*n*-octyl-4’-cyanobiphenyl). The second one, called TBDA (terephthalylidene-bis-4-*n*-decylaniline) also possesses a smectic A phase. 8CB is a nematogenic compound whereas TBDA does not possess any nematic phase; TBDA transits directly into the smectic A phase from the isotropic phase when the temperature is decreased ($T_{I/SmA}∼190^{\circ }C$). Optical measurements were performed with a polarizing microscope (Olympus CX60) coupled to a digital camera which provides images on a computer via an acquisition card. Image Pro Plus (IPP) software permits the recording of the photographs. The temperature control is performed using a Hot Stage HS-2 from Instec which provides a thermo-stabilization accuracy better than $\pm 0.01^{\circ }$ C and a temperature change at a controlled rate, which can be as low as 0.005${}^{\circ }$ C/min. Some droplets of liquid crystal have been deposited between two untreated slides. The liquid crystal is surrounded by a glycerol matrix.

### 2.1. Flower texture

**Figure 2:**
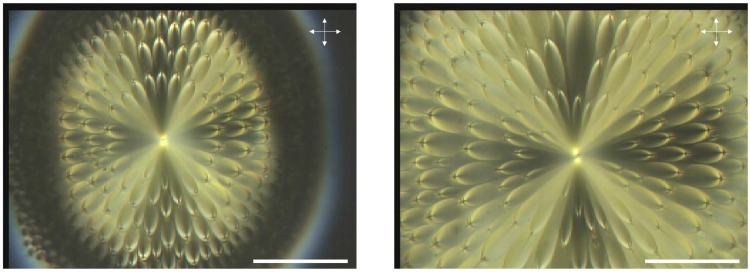
(a): experimental observation of the flower texture in TBDA, bar ∼100μm; (b): experimental observation of the flower texture in TBDA, bar ∼100μm.

The sample has been made with some droplets of TBDA easily deposited on a glass slide without any surface treatment. When the temperature is decreased from the isotropic phase, the smectic A texture appears below $188^{\circ }$C. The texture is not well ordered as it possesses focal conics made up of ellipses and confocal hyperbolae. The temperature has been changed and after several temperature increases and decreases, the sample shows some very regular focal conics texture, the flower texture shown in Figure 2a. What is surprising is the "quasi-isotropic" orientation of the focal conics with some source at the center of the picture. When the temperature is increased or decreased, a focal conic appears from this point and nucleates toward the boundary of the droplet. The untreated glass provides a quasi-homeotropic anchoring but the successive temperature variations induce a specific texture, *i.e.* the flower texture, which exists in the bulk of the sample far from the boundaries. Therefore, the untreated glass doesn’t seem to play any fundamental role in the generation of this texture. In another part of the sample, the texture of Figure 2b is visible: we cannot see the boundaries of the sample keeping the same magnification (objective of the microscope times 50).

### 2.2. Generation texture

**Figure 3:**
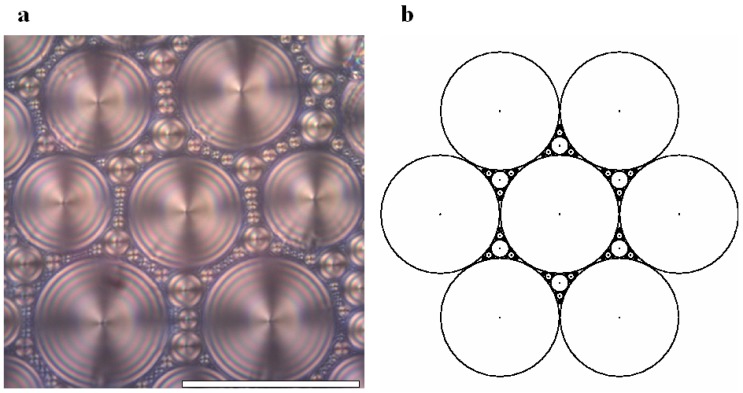
(a): experimental observation of some quasi-hexagonal tiling in 8CB, sample thickness ∼100μm, bar ∼100μm; (b): Apollonius tiling of circles showing 4 generations of sizes using some $C^{++}$ numerical simulation.

**Figure 4:**
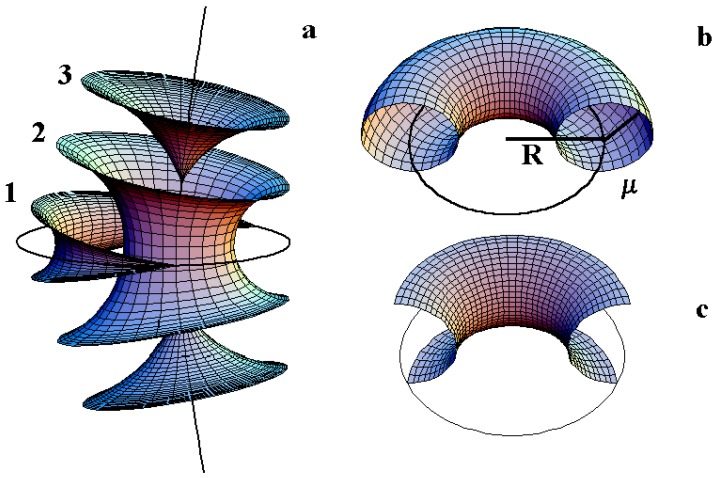
(a): three kind of layers for FCD of the first species; (b): representation of a torus: *R* being the radius of the directing circle and *μ* the radius of the generating circle; (c): representation of a TFCD (Toric Focal Conic Domain) with some negative Gaussian curvature.

8CB smectic liquid crystal has been used surrounded by a glycerol matrix. This glycerol will not mix with 8CB but is used to provide some planar degenerate boundary conditions. Figure 3a represents the generation texture observed under crossed polars microscope which shows the presence of some hexagonal tiling of focal conic domains in the particular case where the ellipses are degenerated into circles and the hyperbolae into straight lines. In this situation, the Dupin cyclide layers become simple torii (see figure 4b). In Figure 3b, some simulation with the Apollonius tiling has been done with 4 generations of circles.

Let us recall the configuration of the layers inside the smectic phase and also the law of corresponding cones (l.c.c).

## 3. The smectic layers

First let us recall the different kind of layers in the case of some usual FCDs. In the figure 4 as described by M. Kleman and O. Lavrentovich [14], three kind of layers in the vicinity of a FCD of the first species can be observed.

-*Layers of type 1* correspond to layers which are singular on the ellipse (two cusps are located on the ellipse).

-*Layers of type 2* correspond to layers which not singular either on the ellipse or on the hyperbola.

-*Layers of type 3* correspond to layers which are singular on the hyperbola (two cusps are located on the hyperbola: one cusp for the upper sheet and another one for the lower sheet).

Note that in the particular case where the ellipse is degenerated into a circle, the Dupin cyclides are simple torii and therefore only two kinds of layers exist: layers of type 2 and layers of type 3. A schematic representation (Figure 4b) recalls two important characteristics of a given torus: the radius *R* of the directing circle and the radius *μ* of the generating circle.

## 4. The Law of Corresponding Cones

**Figure 5:**
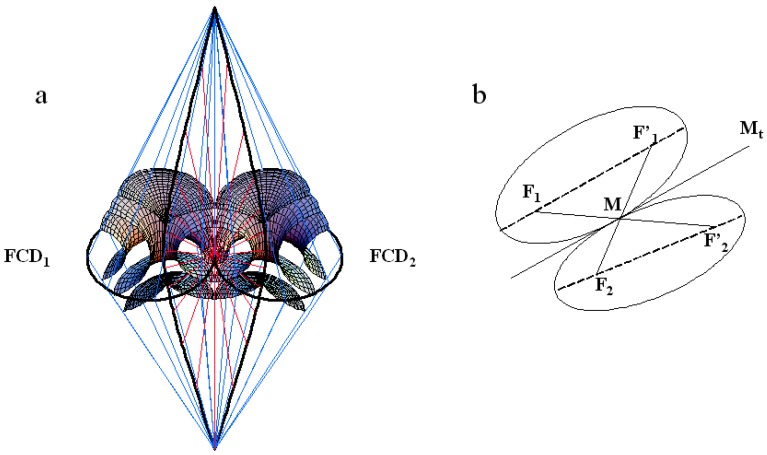
Illustration of the law of corresponding cones (l.c.c) for two tangent ellipses and their confocal hyperbolae ; (a): 3D representation: the two cones which lie on the two ellipses (blue cones) have one common generatrix; likewise for the two cones, which lie on the two hyperbolae (red cones); adapted from [14]; (b): 2D representation: the line joining the visible focus $F_{1}$ of one ellipse to the invisible focus (not physical) of the other $F_{2}^{\prime }$ goes exactly through the tangential point M between the two ellipses; adapted from [23].

In the smectic A phase, the director field should also satisfy to the association rules of focal conic domains (called law of corresponding cones (l.c.c)) first established by G. Friedel [21]. They have been illustrated in particular in the caption of Figure 4 in the paper [22] where the authors wrote that "the cyclides of Dupin mesh smoothly onto spheres. The interface is along a cone of generators; the apex of the cone lies on the hyperbola and is the center of the sphere. The apex of the cones of two adjacent focal domains must coincide at this point." These laws have been recently extended [14]. Two adjacent FCDs should have in common a pair of common generatrices as illustrated in the Figure 5a. The branches of the two hyperbolae intersect in two points corresponding to the "poles" of the hyperbolae (see [21]). These poles both project in the plane of the ellipses onto the same point of the tangent $M_{t}$ common to the ellipses at M. One can see that the two cones with the two poles as apices and which lies on the ellipses have one common generatrix (blue cones). Also, the two physical branches of the hyperbolae belong to the same cone of revolution (red cone) with apex M, axis $M_{t}$. Note that one can go from one layer belonging to $FCD_{1}$ to one corresponding layer belonging to $FCD_{2}$ continuously. Figure 5b shows a 2D representation of the l.c.c. Each ellipse possess two focii: one physical called $F_{i}$, i=1,2 and one non physical called $F_{i}^{\prime }$. The l.c.c reads as follows: the line $F_{1}F_{2}^{\prime }$ goes exactly through the tangency point M of the two ellipses.

Let us come back now to the discussion of the two different textures we have investigated.

## 5. Results and Discussion

### 5.1. Flower texture

#### Model of the experimental texture.

**Figure 6:**
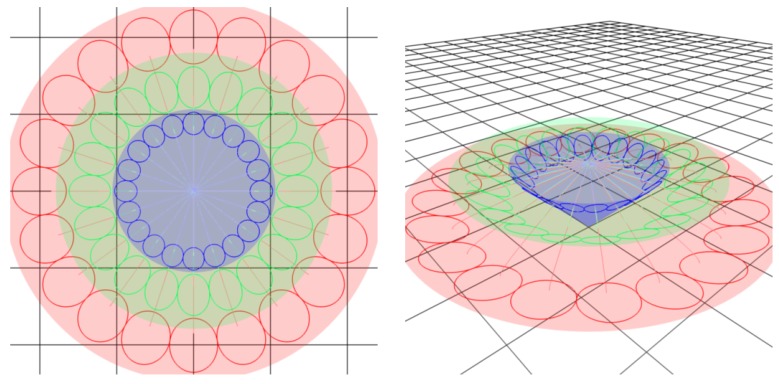
Three cones have been drawn; each cone contains some assembly of mutually tangent ellipses. The *virtual branches* of the hyperbolae have one point of intersection at the apex S of the droplet (a): top view; (b): side view.

The flower texture is particularly symmetric. The ellipses are easily visible. Their confocal hyperbolae appear as straight lines so that they lie in some vertical plane (perpendicular to the horizontal 2D observation plane). The focus with crossed polars optical microscope has been made at the apex of the droplet that we called S in the description. The boundaries of the droplet (see Figure 2a) are completely blurred because of the absence of any cover glass slide. The organization of the ellipses inside the droplet is relatively complicated. The flower texture does not seem to be a classical clustering of FCDs. In fact, the physical hyperbolae seem to be directed outwards of the droplet so that the habit cone of revolution with apex M, axis $M_{t}$ that lies on the physical branches of the hyperbolae is not relevant. On the contrary, we hypothesize that the *virtual branches* of the hyperbolae have one point of intersection at the apex S of the droplet (see Figure 6). Note that in our case, the physical part of the hyperbolae have not been represented; otherwise Figure 6 would have been too much cluttered.

Coming back to the experimental flower texture (Figure 2), if we move from the droplet center to the right along a radius, we see one ellipse then a second one then a third one. Approximately, three ellipses (or four at a few locations) are visible whatever the radius that we consider so that we can imagine three circles relating all the apices of the ellipses located at a constant distance from the center. These circles correspond to cones in the 3D space. Therefore for the model, three cones have been represented: one blue cone corresponds to the ellipses which are located close to the center, a green cone for intermediate ellipse positions and a red cone for the ellipses much closer to the boundary of the droplet. The simulation has been made in the Figure 6a,b with three cones of semi-apex angles of $30^{\circ }$, $45^{\circ }$ and $60^{\circ }$, respectively. These values are in reality close to $90^{\circ }$ but this drawing permits us to explain the model we have in mind. Note also that the differences between the semi-apex angles of the cones are certainly rather small. Looking now to one single cone, all the ellipses which lie on the

generatrices of the cone are mutually tangent. These ellipses respect the law of corresponding cones (l.c.c.). To check this point, let us consider two adjacent tangent ellipses. We check that the line joining the visible focus of one ellipse to the invisible focus (not physical) of the other goes exactly through the tangential point M between the two ellipses. This argument has been used in the book of P. G. De Gennes and J. Prost [23] in the case of the well known "fan-shaped" texture of C. Williams whose associate drawing is given in Figure 1 of reference [22]. We emphasize that in the "fan-shaped" texture, the physical hyperbolae are merging outwards instead of inwards as in the case of the flower texture. Such analysis in terms of application of the l.c.c has been also checked on a very large number of examples [11].

### 5.2. Generation texture

Experimentally, circles are visible in polarized microscopy, so that these two parameters will be important for the description of the layers corresponding to the macroscopic observations.

So the question is: how do the layers fill the space between some hexagonal tiling of circles of some given radius of the first generation of circles. The two parameters of a torus *R* and *μ* (see figure 4b,c) can vary simultaneously but for the sake of clarity, let us first consider the variation of only one of them; the second being fixed. We recall that *R* denotes the radius of the directing circle and that each value of *μ* corresponds to one particular smectic layer.

**Figure 7:**
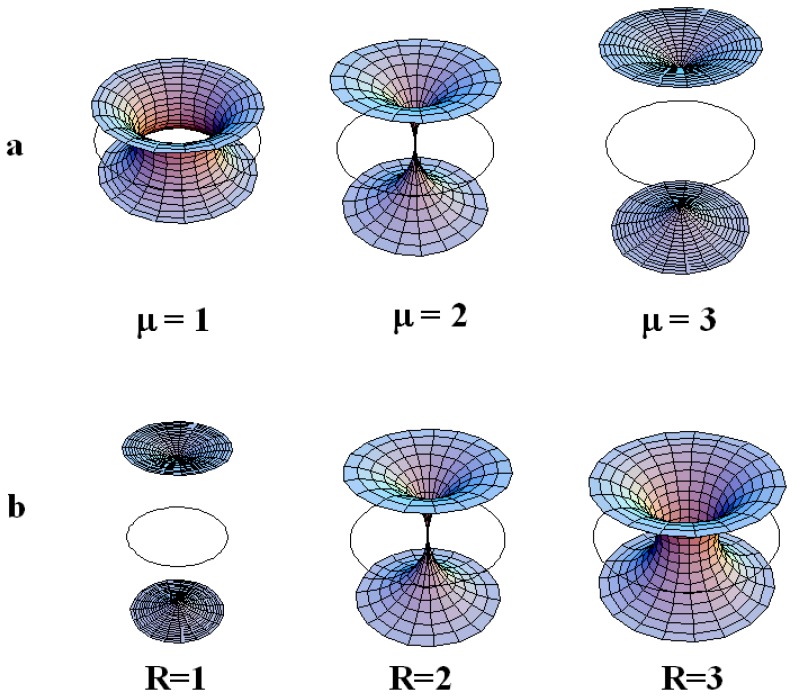
(a): torii with R=2 for different increasing values of μ=1,2,3 from the left to the right. When μ>R, two sheets exist: layer of type 3 takes place instead of type 2; (b): torii with μ=2 for different increasing values of R=1,2,3 from the left to the right. When R>μ, only one sheet exist: layer of type 2 takes place instead of type 3.

#### The variation of *μ* at fixed *R*.

When *R* is fixed, the generation number is fixed because all the radii of circles belonging to a same generation are identical. In the figure 7a where R=2, we see clearly that when μ<R the torii are of type 2, and that when μ>R the torii are of type 3. Therefore, increasing the value of *μ* for a given value of *R* permits to pass from layers of type 2 to layers of type 3 with two sheets. The limit between these two kind of layers is μ=R as represented in Figure 7a, in which R=2 for different varying values of *μ* (μ=1, μ=2 and μ=3). In fact, when one moves in a vertical plane perpendicular to the Apollonius tiling, the layers of type 2 become of type 3 with two sheets (for increasing values of *μ*).

#### The variation of *R* at fixed *μ*.

**Figure 8:**
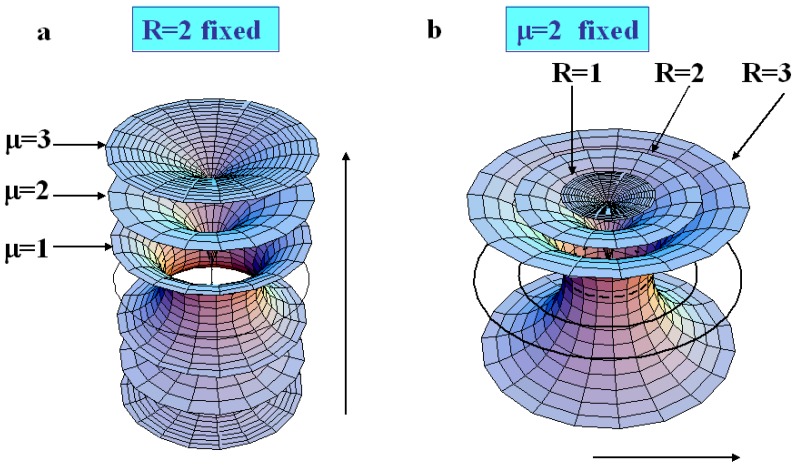
(a): R=2; when *μ* increases layers of type 2 transform into layers of type 3; (b): μ=2; when R increases, layers of type 3 transform into layers of type 2.

When *μ* is fixed, a decrease of *R* corresponds to a change (increase) in the generation number. In the figure 7b where μ=2, we see clearly that for R<μ the torus are of type 3 and that for R>μ, the torus are of type 2. Therefore, increasing the value of *R* for a given value of *μ* permits to pass from layers of type 3 with two sheets to layers of type 2. The limit between these two kinds of layers is R=μ as represented in Figure 7b, in which μ=2 for varying values of *R* (R=1, R=2 and R=3). In fact, when one moves in the plane of the Apollonius tiling from the biggest circles towards the smaller circles, the layers of type 2 become of types 3 with two sheets. These two tendencies are summarized in Figure 8a,b when one moves in the two directions indicated by the arrows.

Our aim is now to represent the layers with different values of *μ* and different values of *R* (several generations, about four are visible under crossed polars optical microscope).

#### The simulation of the smectic layers.

**Figure 9:**
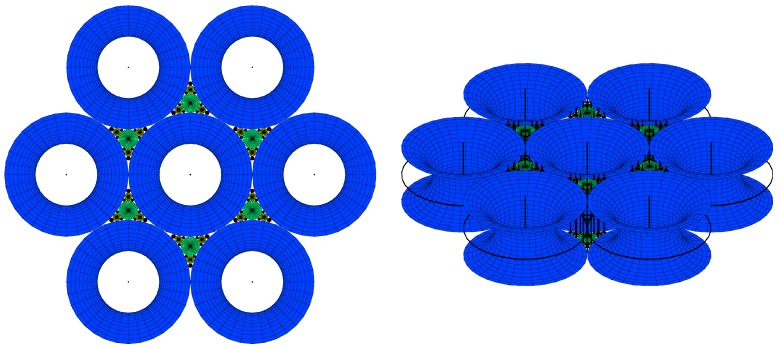
Dupin cyclides simulation: 1rst generation in blue, 2nd in green, 3rd in yellow, 4th in red. (a): Top view ($\theta =0^{\circ }$, $\varphi =90^{\circ }$); (b) some side view ($\theta =60^{\circ }$, $\varphi =30^{\circ }$).

In the experiments on thermotropic liquid crystals in the SmA phase, four generations of FCDs have been observed under optical crossed polars; this is the reason why the simulation has been stopped with four generations although the simulation allows to show up to 10 generations. We do not see higher generations: there is some critical size under which the FCDs do not exist [15]. The macroscopic observations are done when the sample is observed from the top (it means $\theta =0^{\circ }$ and $\varphi =90^{\circ }$). Figure 9a illustrates the simulation corresponding to this experimental situation. Figure 9b corresponds to the visualization of the smectic layers with $\theta =45^{\circ }$ and $\varphi =45^{\circ }$.

**Figure 10:**
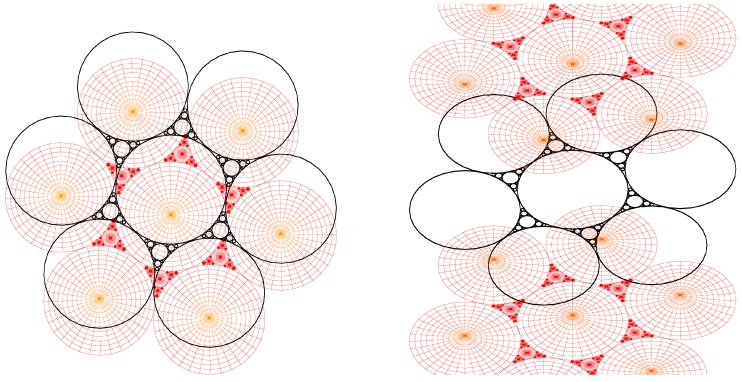
(a): simulation of the smectic layers for one single value of *μ* (μ=3) corresponding to layers of type 3 with two sheets (only one sheet is visible with this point of view); R=0.9, $\theta =10^{\circ }$, $\varphi =80^{\circ }$; (b): the same simulation with another point of view ($\theta =45^{\circ }$, $\varphi =45^{\circ }$).

Each generation corresponds to some fixed value of *R*, so let us try the simulation with some small radius. Our previous discussion shows that if R is small enough, only layers of type 3 occur; this situation has been represented in Figure 10a (only one sheet of each layer is visible) and Figure 10b (the two sheets are visible). Now increasing the starting value of the radius of the first generation of circles *R*, some FCD of type 2 are visible for the first generation (see Figure 10b).

**Figure 11:**
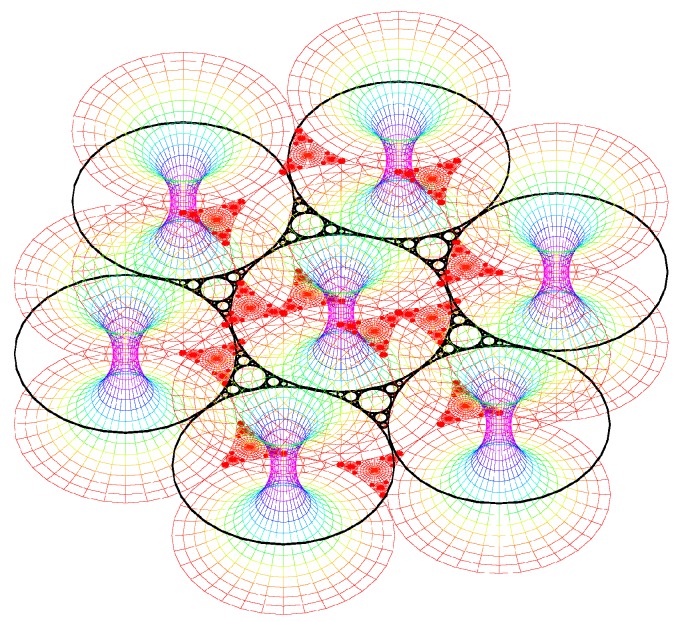
simulation of the smectic layers for one single value of *μ* (μ=3) corresponding to layers of type 3 with two sheets from the second generation of circles; layers are of type 2 for the first generation of circles; R=0.9.

This point leads to a first important result: we can now understand how the continuity between layers of type 2 and 3 occurs: the two sheets of each layer create two hexagonal tilings of the layers: one for the upper sheet and another for the lower sheet (see Figure 11).

**Figure 12:**
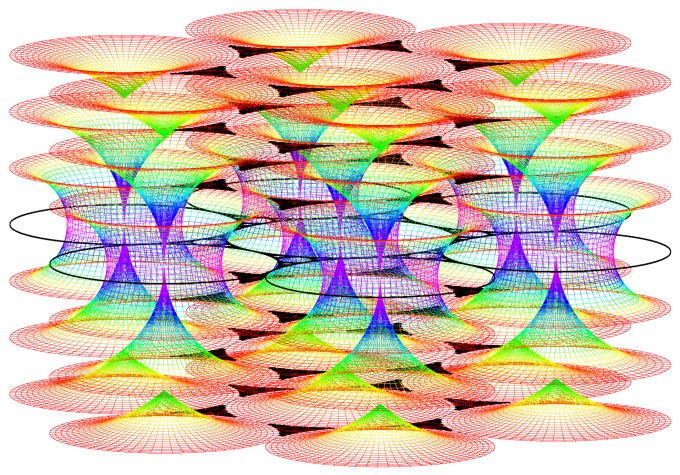
simulation of the smectic layers for three values of *μ* (μ=1; μ=2; μ=3) corresponding to layers of type 3 with two sheets; R=0.9, $\theta =80^{\circ }$, $\varphi =10^{\circ }$.

These two hexagonal tilings are continuously attached with FCDs of type 2 existing for lower generations. Showing now not one value of *μ* for each radius (one layer) but three different values of *μ*, one obtains Figure 12 which allows us to better understand how are the smectic layers close to the singular

lines that are the circles and their cofocal straight lines. The Apollonius tiling corresponding to these singular lines appears always in black color. Finally, some picture of the smectic layers with some visual

**Figure 13:**
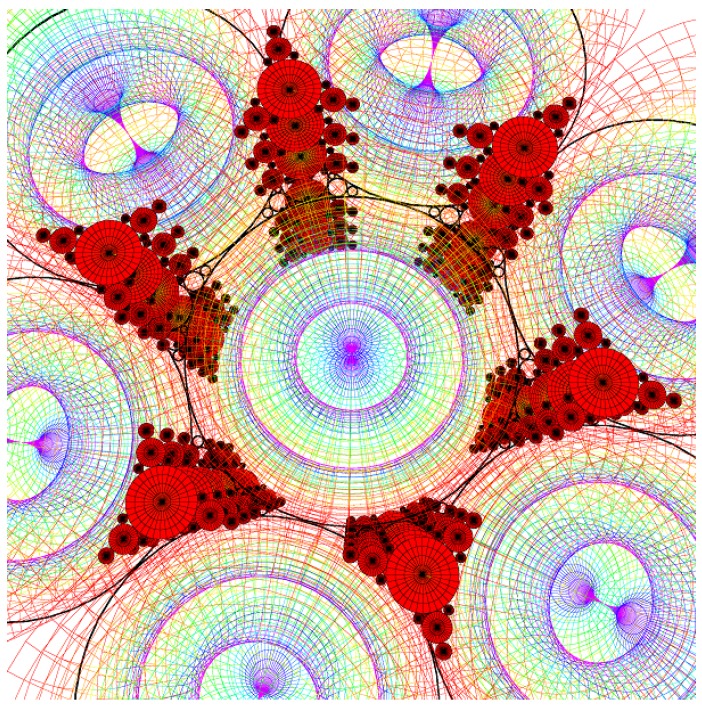
simulation of the smectic layers for different values of *μ*.

effect is presented (see Figure 13).

## 6. Conclusions

Some textures of liquid crystals have been reported in this paper: the flower texture and the generation texture. In the case of the second texture, we successfully represented the smectic layers at the vicinity of the focal conic domains that are circles and straight lines perpendicular to the circles and merging the centers of the circles. The layers of FCD are in general difficult to visualize close to singularities. In the case of a very particular situation (hexagonal tiling of circles), the visualization of the smectic layers allows a better understanding of the topological continuity of the layers. Recently, some imperfections on FCD have been reported [24]. These imperfections are interpreted in terms of interactions of these FCD and dislocations. The visualization of the smectic layers in such a case could be interesting to investigate. Furthermore, this has some great interest in the case of mixture of liquid crystal doped with nanoparticules. The location of the nanoparticules in the core of the FCD as shown recently [25] is another reason to continue the investigation for the representation of the deformed smectic layers at the vicinity of the defects.
